# A prospective, randomized clinical trial comparing bipolar plasma kinetic resection of the prostate versus conventional monopolar transurethral resection of the prostate in the treatment of benign prostatic hyperplasia

**DOI:** 10.4103/0256-4947.57163

**Published:** 2009

**Authors:** Christopher Ho Chee Kong, M. Fadzli Ibrahim, Zulkifli Md Zainuddin

**Affiliations:** From the Division of Urology, Department of Surgery, University Kebangsaan Malaysia Medical Centre, Kuala Lumpur, Malaysia

## Abstract

**BACKGROUND AND OBJECTIVE:**

For treatment of benign prostatic hyperplasia (BPH), Plasma Kinetic loop Resection of the Prostate (PKRP) is an alternative to conventional monopolar transurethral resection of prostate (TURP). We compared outcomes with the two treatments in a randomized trial.

**PATIENTS AND METHODS:**

Over a one-year period, we randomly assigned patients with an indication for surgery for BPH and who met inclusion criteria to treatment with either PKRP or TURP. We measured prostate volume by transrectal ultrasound, relief of bladder outlet obstruction, operative time, decline in serum sodium and hemoglobin, weight of resected prostatic chips, duration of catheterization and hospital stay. Patients were evaluated one month after discharge for obstructive symptoms. Complications were also recorded.

**RESULTS:**

Of 102 patients enrolled, 51 underwent PKRP and 51 underwent TURP. Relief of obstructive symptoms and mean operative time showed no statistically significant difference. The PKRP group had a smaller decline in hemoglobin than the TURP group (0.6 g/dL vs 1.8 g/dL, *P*=.01), a lower reduction in serum sodium levels (1.03 mmol/L vs 5.01 mmol/L, *P*=.01), a shorter catheterization time (37.2 hours versus 57.7 hours, *P*=.03) and a shorter hospital stay (1.5 days versus 2.6 days, *P*=.02). One patient in the bipolar PKRP group needed recatheterization versus four patients in the TURP group.

**CONCLUSION:**

PKRP reduces morbidity with an outcome similar to conventional monopolar TURP in the treatment of BPH.

Transurethral resection of prostate (TURP) is currently the gold standard for surgical treatment of benign prostatic hyperplasia (BPH) as this procedure results in the best improvement in symptoms and urine flow rate.^1^ However, this procedure is not free of complications. Mebust et al reported an 18% morbidity rate after TURP and a metanalysis by the BPH Guideline Panel showed that the morbidity rate associated with TURP ranges from 7% to 43%.^2^ This has brought about the emergence of alternative modes of treatment for BPH with the aim of reducing complications, morbidity, hospital stay and cost. plasma kinetic loop resection of the prostate (PKRP) is one such option. It is a relatively new method that has been reported to produce results comparable with conventional TURP.^3^ PKRP uses bipolar diathermy as compared with the conventional monopolar diathermy in TURP. We compared the outcome in the relief of bladder outlet obstruction (BOO), complications, operative time and hospital stay, between these two instruments.

## PATIENTS AND METHODS

From November 2007 to October 2008, patients with an indication for surgery for BPH were enrolled in a prospective, randomized clinical trial comparing bipolar PKRP versus conventional TURP. The indications for surgery were either moderate to severe Lower Urinary Tract Syndrome that had failed medical treatment, had complications of BOO, or catheter dependency. Exclusion criteria were American Society of Anethesiologists score more than II, use of a pacemaker, suspected or known prostate cancer, concurrent bladder stone and previous bladder neck surgery. Approval was obtained from the Universiti Kebangsaan Malaysia Research and Ethics Committee.

All patients enrolled in the study were given a thorough explanation of both modes of treatment and informed consent was taken prior to randomization. Allocation concealment was done via sequentially numbered, opaque, sealed envelopes. Computerized random-number generator was then used to select an envelope for each patient. A nurse not involved in this study then read the content of the envelope and assigned the appropriate method of surgery. All patients were blinded to the type of treatment method. Preoperatively, BOO was assessed using the International Prostate Symptom Score (IPSS) score, peak flow volume (Qmax) and post-void residual urine volume (PVR) for the severity of the BPH. Blood tests were sent for hemoglobin (Hb) and serum sodium levels.

Both surgeries were performed under spinal anaesthesia by two consultants, both with 5 years experience as consultant urologists. Prostate volumes were measured using transrectal ultrasound. The surgical cutting electrode instruments were of similar design and the surgical technique was also similar in both methods. Storz Fr27 continuous flow resectoscopes with loop electrodes were used in both procedures. Plasma kinetic resection of the prostate was performed using bipolar plasma kinetic tissue management electrosurgical system (Gyrus Medical Ltd, Buck, UK) with the power setting at 240W for vaporization and 60W for coagulation. Conventional TURP was carried out using Pfizer (Valleylab-Monopolar Diathermy) electrosurgical instrument system with the setting at 60W for coagulation and 120W for cutting. In bipolar PKRP, electrical energy is delivered via a bipolar generator. An ionized plasma pocket is created that allows resection and vaporization of the tissue along with haemostasis. Both the active and return electrode are contained within the instrument. Saline solution is used as an electrolytic medium to conduct the electrical energy from the active to the return electrode. In contrast, conventional TURP is performed using monopolar electric current from the electrosurgical unit which flows from the active electrode (the wire loop), through the patient, to an electrosurgical unit grounding pad. Glycine, which is a non-conducting fluid, is used for irrigation.

Operative time was recorded as the time from the introduction of the cystoscope until the insertion of 22F 3-way Foley catheter for normal saline irrigation.

At the end of the surgery, the weight of the prostate chips were measured. Signs and symptoms of transurethral resection (TUR) syndrome were also assessed clinically. Hemoglobin (Hb) and serum sodium levels were reassessed at one hour and 24 hours post-operatively. Bladder irrigation with normal saline was continued until there was no more hematuria. The catheter was then removed within 24 hours of clear urine except in patients who developed complications such as hematuria and clot retention. In those patients, a rigid cystoscope was performed, bleeders diathermised and the catheter was then removed after 24 hours of clear urine. Changes in hemoglobin, hematocrit, serum sodium level, catheterization time, duration of hospital stay and other complications were recorded. Patients were reviewed one month later for evaluation of the effectiveness of the treatment in the relief of BOO as well as to document any complications.

Sample size was calculated a priori with the alpha level set at 0.05, an anticipated effect size (Cohen's *d*) of 0.5 and a desired statistical power level of 0.8. The required sample size per group was 51. A paired *t*-test and Mann-Whitney test were used for the analysis of the variables and categorical data. Differences were considered significant at a *P* value less than.05.

## RESULTS

The 102 patients enrolled in the study were randomized into groups of 51 patients each. The median age was 68 years (range, 41-82 years old) (Table 1). The study population included 52 Malays (51.0%), 43 Chinese (42.1%), and 7 Indians (6.9%). The main indication for surgical intervention was failed medical treatment (55%). Pre-operative assessments of the severity of BPH are shown in Table 1. The mean postoperative IPSS score was 6.1 in the PKRP group and 6.5 in the TURP group (*P* =.60). Assessment of mean peak flow volume (Qmax) value at one month post-operation showed a significant improvement to 16.6 mL/s in the PKRP group (*P* =.02 and 17.6 mL/s in the TURP group (*P*=.01). Statistical analysis between the mean difference of peak flow volume (Qmax, pre- and postoperatively) against the type of surgery showed no significant difference (*P*=.29). Mean post-void residual (PVR) volume improved postoperatively to 21.2 mL in the PKRP group and to 24.3 mL in the TURP group (*P*=.17). Differences in the decline in hemoglobin and serum sodium and in catherization time and hospital stay between the groups were statistically significant (Table 2).

**Table 1:** Summary of variables measured.

Variable	PKRP Mean (SD)		Monopolar Mean (SD)	
	Pre-op	Post-op	Pre-op	Post-op
Age (years)	68.44 (7.33)		68.53 (6.69)	
TRUS (mL)	41.8 (9.80)		43.1 (10.94)	
IPSS	23.3 (4.77)	6.10 (1.47)	23.9 (4.32)	6.50 (1.33)
QOL	4.47 (0.81)	1.98 (0.60)	4.51 (0.76)	1.80 (0.37)
Q max (mL/s)	4.99 (1.48)	17.64 (2.86)	4.60 (1.61)	16.51 (2.53)
PVR (mL)	107 (28.01)	24.21 (5.76)	103 (24.83)	21.37 (6.62)
Hb (g/dL)	12.67 (2.04)	12.10 (1.56)	12.82 (1.16)	12.60 (1.67)

IPSS=International Prostate Scoring System; QOL=quality of life score; Qmax=maximum flow rate; PVR=post-void residual urine volume; TRUS=transrectal ultrasound; SD=standard deviation; Hb=hemoglobin

**Table 2:** Summary of significant results.

Variable	PKRP Mean(SD)	TURP Mean(SD)	*P*
Hemoglobin decline (g/dl)	0.6 (1.48)	1.8 (1.41)	.01
Serum sodium decline (mmol/l)	1.03 (2.36)	5.01(1.77)	.01
Catheterization time (hours)	37.2 (15.03)	57.7 (17.31)	.03
Hospital stay (days)	1.5 (0.88)	2.6 (0.92)	.02

SD=standard deviation; PKRP=plama kinetic resection of prostate; TURP=transurethral resection of prostate

**Figure 1:**
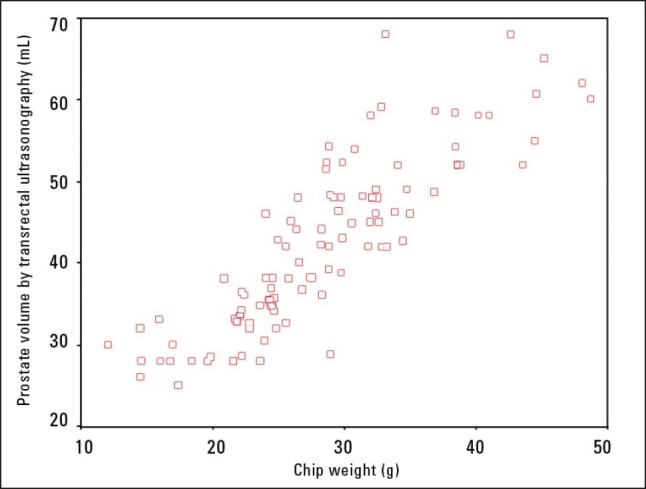
Correlation between transurethral ultrasound prostate volume (mL) and prostatic chip weight resected (g).

Reassessment one month after surgery showed no significant differences in patient satisfaction in the relief of BOO. The average pre-operative prostate volume was 41.8 mL in the PKRP group and 43.1 mL in the TURP group. The mean chip weight of the resected prostate glands were 24.7 g in the PKRP group and 26.6 g in the TURP group (*P*=.446), meaning that 62.4% and 59.1% of the glands, respectively, were resected (Figure 1).

There were no major complications in either group. No transurethral resection (TUR) syndrome was noticed clinically in any of the patients. Only one patient in the PKRP group needed recatheterization whereas four patients in the TURP group required recatheterization. Of those, two patients failed catheter removal and were discharged with an indwelling catheter. Two of the patients in the TURP group also needed prolonged hospitalization (one patient stayed five days and the other one needed seven days-post operative hospital stay) due to secondary hemorrhage that required blood transfusion.

## DISCUSSION

Standard transurethral resection uses basic electrosurgical principles to achieve effective and rapid removal of prostate tissue. Recently, plasmakinetic electrovaporization uses these principles to combine vaporization and resection in a simultaneous action that achieves the desired effects of the standard conventional TURP.^1^ There have been a number of studies reporting improvements in objective parameters compared to conventional TURP.^1^–^9^ In the present trial, the main objective of evaluating the outcome in relief of BOO between the two groups did not show a statistically significant difference (parameters measured were IPSS score, Qmax and PVR different pre- and postoperatively). This finding was also observed in a previous series.^4^,^6^ This may indicate that the plasmakinetic method has comparable effect in relief of bladder obstruction symptoms (equivalent effect to standard conventional group).

The mortality associated with TURP is very low (less than 0.25%). However, the procedure has complications, such as blood loss, that need blood transfusion (up to 8%) and transurethral resection (TUR) syndrome (up to 2%).^6^ The sharp cutting action using plasmakinetic electrovaporization and resection using a loop electrode (as in the Gyrus system) should prevent these complications, improving homeostasis during the procedure and reducing the risk of hyponatremia (normal saline instead of glycine was used as the irrigation fluid).^6^,^10^–^13^ The bipolar electrosurgical equipment simultaneously vaporizes tissue during resection, which controls bleeding as it effectively and accurately seals all bleeding points.^8^,^10^,^14^,^15^ The plasmakinetic system, however, uses a smaller loop compared with the conventional TURP, which requires more strokes for every unit volume of prostate resected.^9^,^16^–^18^ This explains the insignificant operative time difference between the two groups despite the advantage of better hemostasis.^9^,^17^,^18^

The systemic absorption of glycine contributes to TUR syndrome.^11^,^12^ The risk increases from 0.7% to 2% if the resection time is longer than 90 minutes and for a larger prostate (>45 g).^11^,^12^ Using the plasmakinetic system reduces the risk as the irrigation solution used is normal saline. In our series, TUR syndrome was not observed in both groups. Even though the serum sodium drop in conventional monopolar group showed statistical significance (*P* value <.05), it was only detected biochemically and was not severe enough to cause clinical manifestation of TUR syndrome.

Blood loss is the most frequent postoperative complication of TURP. In our cohort, the mean decrease in hemoglobin level at 24 hours after surgery was lower in the plasmakinetic bipolar group and this was statistically significant (*P* <.05). There was no major bleeding episode in plasmakinetic bipolar group, while in the conventional monopolar group two patients developed secondary hemorrhage that needed blood transfusion. This effect of better homeostasis was also seen in many previous studies^4^,^6^ except that some did not show statistical significance change.

The patients treated by the plasmakinetic bipolar method had their catheter removed at mean of 37.2 hours, which was earlier compared to the conventional monopolar group (mean 57.7 hours). This advantage was observed mostly due to a better hemostatic effect exerted by the plasmakinetic method. This was obviously followed by a shorter post-operative hospital stay in the plasmakinetic bipolar group (mean 1.5 days) compared to the conventional monopolar group (mean 2.6 days) thus translating into a reduction of cost (earlier discharge from hospital).

In conclusion, PKRP is comparable to TURP in terms of efficacy in the relief of BOO. It has the further advantage of better hemostasis as proven by less blood loss, no significant reduction of serum sodium, less catheterization time and a shorter hospital stay. It may also enable prostate resection as a day case in selected cases. These data are promising, but a longer follow up and larger series are needed to compare the late complications such as urethral stricture, bladder neck stenosis and retrograde ejaculation, before the bipolar PKRP method becomes universally accepted for managing BPH.
